# Impaired Function of the Calcium-Dependent Protein Kinase, *OsCPK12*, Leads to Early Senescence in Rice (*Oryza sativa* L.)

**DOI:** 10.3389/fpls.2019.00052

**Published:** 2019-02-04

**Authors:** Beifang Wang, Yingxin Zhang, Zhenzhen Bi, Qunen Liu, Tingting Xu, Ning Yu, Yongrun Cao, Aike Zhu, Weixun Wu, Xiaodeng Zhan, Galal Bakr Anis, Ping Yu, Daibo Chen, Shihua Cheng, Liyong Cao

**Affiliations:** ^1^Key Laboratory for Zhejiang Super Rice Research and State Key Laboratory of Rice Biology, China National Rice Research Institute, Hangzhou, China; ^2^College of Agronomy, Gansu Agricultural University, Lanzhou, China; ^3^Nanchong Academy of Agricultural Sciences, Nanchong, China; ^4^Rice Research and Training Center, Field Crops Research Institute, Agriculture Research Center, Kafr El Sheikh, Egypt

**Keywords:** rice (*Oryza sativa* L.), early senescence, *es4*, *OsCPK12*, calcium-dependent protein kinase

## Abstract

Premature leaf senescence affects plant yield and quality, and numerous researches about it have been conducted until now. In this study, we identified an early senescent mutant *es4* in rice (*Oryza sativa* L.); early senescence appeared approximately at 60 dps and became increasingly senescent with the growth of *es4* mutant. We detected that content of reactive oxygen species (ROS) and malondialdehyde (MDA), as well as activity of superoxide dismutase (SOD) were elevated, while chlorophyll content, soluble protein content, activity of catalase (CAT), activity of peroxidase (POD) and photosynthetic rate were reduced in the *es4* mutant leaves. We mapped *es4* in a 33.5 Kb physical distance on chromosome 4 by map-based cloning. Sequencing analysis in target interval indicated there was an eight bases deletion mutation in *OsCPK12* which encoded a calcium-dependent protein kinase. Functional complementation of *OsCPK12* in *es4* completely restored the normal phenotype. We used CRISPR/Cas9 for targeted disruption of *OsCPK12* in ZH8015 and all the mutants exhibited the premature senescence. All the results indicated that the phenotype of *es4* was caused by the mutation of *OsCPK12*. Overexpression of *OsCPK12* in ZH8015 enhanced the net photosynthetic rate (*P*_n_) and chlorophyll content. *OsCPK12* was mainly expressed in green organs. The results of qRT-PCR analysis showed that the expression levels of some key genes involved in senescence, chlorophyll biosynthesis, and photosynthesis were significantly altered in the *es4* mutant. Our results demonstrate that the mutant of *OsCPK12* triggers the premature leaf senescence; however, the overexpression of *OsCPK12* may delay its growth period and provide the potentially positive effect on productivity in rice.

## Introduction

Senescence is a series of deteriorative processes including death and decomposition of cell, degradation of organization and organ, and the aging of the life function. Leaf senescence as a type of programmed cell death (PCD) is a critical process for the adaptability of plants (Himelblau, 2000). As a complex physiological process, leaf senescence is not only influenced by external environment such as temperature, light, drought, nutrient deficiency, wounding, pathogen infection, etc. (Yang et al., 2011), but also affected by internal genetic factors such as developmental stage and phytohormone levels (Yang et al., 2016). Premature leaf senescence has a direct impact on crop yields by changing the duration of photosynthesis, and modifying the nutrient remobilization efficiency and harvest index (Wu et al., 2012). As one of the major food crops, rice feeds nearly half of the world’s population, but rice leaf premature senescence often results in the reduction in yield and quality. On the contrary, delayed leaf senescence shows potentially positive effects on rice productivity. Therefore, understanding the molecular mechanism of leaf senescence is important for breeders in raising rice production and quality.

Many rice leaf senescence-associated genes have been identified from different plant species. These genes can be divided into different categories according to the metabolic pathways. One type of genes synthesize chloroplast and degrade chlorophyll including *NYC1*, *NOL*, *OsPAO*, *OsRCCR1*, *OsSGR*, *V1*, *V2* and so on (Jiang et al., 2007; Kusaba et al., 2007; Kusumi et al., 2011; Tang et al., 2011). The second type involved in synthesizing, degrading and transporting proteins including *GnT1*, *OsSAG12*, *Osh69*, *TDC1*, *TDC2*, etc. (RH et al., 2004; Kang et al., 2009; Fanata et al., 2013; Singh et al., 2013). The third type of genes involve in the hormone signaling pathway. For example, *OsFBK12*, *OsSAMS1*, *ein2,* and *EIN3* involved in ethylene signaling pathways (Alonso et al., 1999; Chen et al., 2013; Li et al., 2013). PCD-related genes belong to the fourth type of genes, which play an important role in the process of aging. *RLS1* encodes a previously uncharacterized NB-ARM protein regulating PCD during leaf senescence (Jiao et al., 2012). In addition, three transcription factor families including NAC, WRKY, and TCP involved in leaf senescence. *OsY37*, an NAC transcription factor gene positively regulates the heading date and senescence during the reproductive phase in rice (El Mannai et al., 2017). Although great progress has been made on rice leaf senescence research, rice leaf senescence is a complex process which involves many genes and metabolic pathways. The molecular mechanisms of leaf senescence are still remain unclear and further work about it should be done.

Calcium-dependent protein kinases (CPKs) as a type of calcium sensor, which contain a kinase catalytic domain and an autoinhibitory “junction” domain, followed by a calmodulin-like, a regulatory domain (Hrabak et al., 2003). CPKs perform multiple biological function in plants such as senescence and cell death, hormones signal transduction, stress and defense responses, growth and development, carbon and nitrogen metabolism, formation of cytoskeleton, regulation of ion channels, etc. (Lei et al., 2007). For example, NtCDPK1 and NtRpn3 are expressed in rapidly growing tissues, and knocking down protein expression led to severe growth defects with abnormal cell morphology and premature cell death of newly developing leaves (Simeunovic et al., 2016). A 57 kD calcium-dependent protein kinase (CDPK) molecule has multiple sites for autophosphorylation, and the changes in *in vivo* autophosphorylation status of the 57 kD CDPK induced by ST (Senescence-inducing treatment) may play an important role in regulating the catalytic activity of leaf senescence in Soybean Primary Leaves (Wang et al., 2001). CPK10 plays important roles in ABA and Ca^2+^-mediated regulation of stomatal movements possibly by interacting with HSP1 (Zou et al., 2010). OsCPK21 is involved in the positive regulation of the signaling pathways that are involved in the response to ABA and salt stress (Asano et al., 2011). OsCPK4 is a positive regulator of the salt and drought stress responses in rice via the protection of cellular membranes from stress-induced oxidative damage (Campo et al., 2014). AtCPK6 is functionally redundant and a positive regulator involved in the tolerance to salt/drought stress in Arabidopsis (Xu et al., 2010). Rice SPK, a calcium-dependent protein kinase, is expressed uniquely in the endosperm of immature seed, and SPK is involved in the activation of Suc synthase that may be important for supplying substrates for the biosynthesis of storage products (Asano et al., 2002). CPK11 and CPK24 together mediate the Ca^2+^-dependent inhibition of K^+^ channels and participate in the regulation of pollen tube growth in Arabidopsis (Zhao et al., 2013). The phosphorylation of Sucrose phosphate synthetase (SPS) and nitrate reductase (NR) might be conducted by one CPK (Chung et al., 1999). Putnam’s study indicated that CPK was bound with actin filament in pollen tube and internodal cell (Putnam et al., 1989). CPKs also regulated ion channels in guard cell vacuolar membrane (Pei et al., 1996) and inward K^+^ channel of plasmalemma (Li et al., 1998; Berkowitz et al., 2000). Besides CPKs are related with stomatal movement, metabolism of enzymes, membrane transport and many other biologic functions and more and more functions of CDPK family members will be identified. Multifunctional CPKs are found in both vascular and non-vascular plants (Harmon et al., 2001). There are 34 genes in Arabidopsis and 31 genes in rice encoding CPKs, respectively (Cheng et al., 2002; Ray et al., 2007).

In this study, we isolated and characterized an early senescent (*es4*) mutant in rice which displayed early leaf senescence phenotype along with lower seed setting rate and 1000-grain weight, less ROS, lower photosynthetic capacity and Chlorophyll content. Map-based cloning and sequencing analysis showed that the loss of eight bases led to a frame-shift mutation in *OsCPK12* which encoded a calcium-dependent protein kinase. The following functional validation demonstrated that the mutation of *OsCPK12* not only led to leaf senescence but also influenced the yield-related traits in rice.

## Materials and Methods

### Plant Materials

The early senescence 4 (*es4*) mutant was isolated from a Co^60^ γ-ray treatment rice mutant library of the *indica* rice cultivar, ZH8015. The F_2_ population, derived from cross ZH8015 and 02428 (a *japonica* rice cultivar) was used for genetic analysis and mapping of *es4*. All the plants were grown in a paddy field under natural conditions in Hangzhou, Zhejiang province and in Lingshui, Hainan Province, China. The lower leaves of *es4* firstly showed senescent phenotype at 60 days post-sowing (dps). In the F_2_ population, the plants with leaf senescence phenotype were used for fine genetic mapping.

### Hydrogen Peroxide, Cell Death and Superoxide Anion Detection

Qualitative analysis of hydrogen peroxide (H_2_O_2_), cell death and superoxide anion was conducted by 3,3-diaminobenzidine (DAB), evans blue (EB) and nitroblue tetrazolium (NBT) staining as previously reported by Thordal-Christensen et al. (1997); Ramel et al. (2009) and Kong and Li (2011). The third leaves of ZH8015 and *es4* mutant were taken from the plants grown in the paddy field at 70 dps.

### TUNEL Assays

The terminal deoxynucleotidyl transferase-mediated dUTP nick end labeling (TUNEL) assays were performed using a Fluorescein In Situ Cell Death Detection Kit (Roche) following the manufacturer’s instructions. The methods were used for sectioning and fluorescence labeling as previously reported by He et al. (2018). The green fluorescence of fluorescein and blue fluorescence of DAPI were analyzed using a Carl Zeiss LSM 710 laser-scanning confocal microscope (Göttingen). The third leaves of ZH8015 and *es4* mutant were taken from the plants grown in the paddy field at 70 dps.

### Measurement of Chlorophyll Content and Photosynthetic Rates

The chlorophyll was extracted from the third upper leaves at 70 dps and determined according to Porra et al. (1994). The OD values under the wavelengths of 450, 663, and 646 nm were obtained with a DU800 visible spectrophotometer (BACKMAN COULTER DU800, United States). The content of Chlorophyll a and b were analyzed according to the method as described by Wellburn (1994).

At 9:00–11:00 on a sunny day, the net photosynthetic rate (*P*_n_) of third upper leaves was determined by the portable photosynthesis measurement device LI-6400 (Li-COR, Lincoln, NB, United States) with 1200 μmol protons (m^2^.s) intensity and 500 μmol⋅s^-1^ airflow rate under field conditions at 70 dps. All experiments were repeated with three biological replicates. Student’s *t*-test was conducted by EXCEL2013 and multiple comparison was conducted by SAS 9.0.

### Measurement of Enzymatic Activity and Senescence-Related Parameters

The activities of catalase (CAT), superoxide dismutase (SOD), peroxidase (POD), ascorbate peroxidase (APX) and the content of soluble protein (SP), H_2_O_2_ and malondialdehyde (MDA) were determined using commercial assay kits from Nanjing Jiancheng Bioengineering Research Institute (China). The third upper leaves of ZH8015 and *es4* mutant were taken from the plants grown in the paddy field at 70 dps. Phenotypic values are the means of three biological replicates. Statistical analysis was conducted by EXCEL2013.

### Observation of the Chloroplast Structure by Transmission Electron Microscopy

At 70 dps, the third leaves from ZH8015 and *es4* were fixed using 2.5% glutaraldehyde in 0.2 M phosphate buffer (pH 7.0) for more than 16 h at 4°C. After three briefly rinsed in the phosphate buffer, the samples were treated with 1% (w/v) OsO_4_ in phosphate buffer (pH 7.0) at 4°C overnight and then dehydrated in a graded series of ethanol [30, 50, 70, 85, 95, and 100% (v/v)]. Ethanol was subsequently replaced by a series of Spurr’s resin dilutions [25, 50, 75, and 100% (v/v)] for approximately 15–20 min at each step. The samples were placed in a 1:1 mixture of alcohol and 90% acetone for 20 min at room temperature. Next, the samples were transferred into 90% acetone for 20 min and then into 100% acetone for dehydration treatment three times, 15 min every time. After dehydration treatment, the samples were transferred into a final Spurr resin mixture overnight. The specimens were then placed in capsules with embedding medium and heated at 70°C for 9 h. The specimen sections were stained using uranyl acetate and alkaline lead citrate for 15 min each and observed using a TEM (Model H-7650) at the institute of Agriculture and Biotechnology, Zhejiang University.

### Map-Based Cloning of *ES4*

F_1_ plants derived from the cross between ZH8015 and 02428, were grown in the paddy field at Lingshui experimental Station of CNRRI in 2014 for determining dominance or recessiveness of *ES4*, and the F_2_ population was used for segregation analysis. 77 F_2_ individual plants with the mutant phenotype were used for preliminary mapping of the *ES4*. The initial mapping was conducted using 145 SSR and InDel markers scattered across 12 chromosomes in rice. To further narrow down the *ES4* region, many new InDel markers were designed using Primer Premier 5.0 after comparing the sequences between Nipponbare and 93-11 in Gramene^1^. All the primers were synthesized by TsingKe technology company (Hangzhou, China). The marker information was presented in Supplementary Table S2. The DNA was extracted by cetyltriethyl ammonium bromide (CTAB) (Murry and Thompson, 1980). The PCR was performed using 2 × Taq PCR Mix from TsingKe technology company (Hangzhou, China). The reaction system and program of PCR referred specification. PCR products were visualized on 8.0% non-denaturing polyacrylamide gel using silver staining.

### Plasmid Construction and Plant Transformation

For functional complementation of the *es4* mutant, a 6745 bp genomic DNA that contains the *ES4* coding region along with the upstream sequence and downstream sequences was amplified from wild-type ZH8015 by PCR using the ES4-COM primer (Supplementary Table S2), and then was introduced into the binary vector pCAMBIA1300 using the In-Fusion HD Cloning Kit (Clontech, Japan). We generated a 23 bp target sequence (5′-TCGACCGCATCACGGCCAAGGGG-3′) in CRISPR-P^2^. The target sequence was cloned into BGK03 vector which was digested by ECO31I and connected by T4-ligase. BGK03 vector (Biogle Company, China) contains a codon-optimized Cas9 driven by a maize strong promoter (UBI), the *OsU6* promoter and gRNA scaffolds of Cas9 expression backbone vector (Li et al., 2016). To create the overexpression vector construct, a 1754-bp 5′ upstream region of the *ES4* gene was amplified by PCR using *ES4*-OE primers (Supplementary Table S2), and the sequence was cloned into pCAMBIA2300 vector which was digested by SmaI and XbaI. All the constructs were checked by DNA sequencing. All vectors were transformed into ZH8015 or *es4* mutant via the *Agrobacterium tumefaciens-*mediated transformation method. We used T3 transgenic plants for phenotypic investigation and physiological study.

### RNA Extraction and qRT-PCR

Total RNA was isolated from rice organizations, including roots, stems, leaves, leaf sheaths, and panicles using the RNAprep Pure Plant Kit (TIANGEN, Beijing). The cDNA was converted from total RNA using ReverTra Ace qPCR-RT Master Mix (Toyobo, Japan). qRT-PCR was performed using SYBR premix Ex Taq II (Takala, Japan) in the LightCycler 480 II (Roche, Sweden) by the Methods and procedures of the manufacturer’s instructions. *OsActin1* was used as control. The primers used for RT-PCR are listed in Supplementary Table S3. The 2^-ΔCT^ and 2^-ΔΔCT^ method was used to analyze the relative transcript levels in gene expression. Values are the means of three biological replicates. *T*-test and multiple comparison were conducted by EXCEL2013 and SAS 9.0, respectively.

## Results

### Phenotype of the *es4* Mutant

**FIGURE 1:**
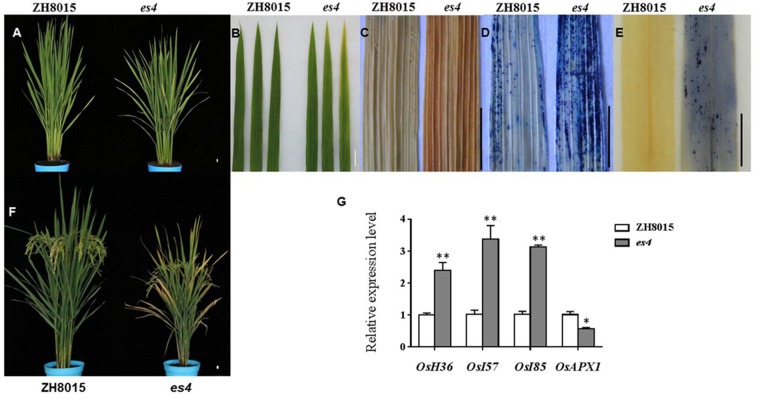
Phenotypic characteristics of *es4* and the wild-type ZH8015. **(A)** Phenotype of ZH8015 and *es4* mutant at 70 dps. **(B)** A close up of the upper three leaves in ZH8015 and *es4* mutant. **(C)** DAB staining. **(D)** Evans blue staining. **(E)** NBT staining. **(F)** Plant of ZH8015 and *es4* at the mature stage. **(G)** The expression levels of senescence-related genes (mean ± S.D., *n* = 3). ^∗^ significance at *P* < 0.05, ^∗∗^ extremely significance at *P* < 0.01. Scale bar = 2 cm.

The *es4* mutant exhibited a premature senescence leaf, dwarf and lower yield phenotype. There was no obvious phenotypic difference between *es4* and ZH8015 at the early developmental stage. The tips and margins of the lower leaves of *es4* became yellow approximately at 60 dps (Figure 1A,B); all leaves of the *es4* became yellow and senescent at the grain-filling stage while most leaves of wild type were still green at the same time (Figure 1F). The plant height, spikelet number per panicle, seed setting rate and 1000-grain weight were remarkably decreased in the *es4* mutant. Compared with ZH8015, spikelet number per panicle, seed setting rate and 1000-grain weight of es4 decreased by 16.14, 9.45, and 8.62%, respectively (Table 1). These results indicated that the early senescent leaves in *es4* would negatively affect the grain yield.

**Table 1:** Comparison of agronomic traits between wild type ZH8015 and mutant *es4* (mean ± SD, *n* = 10).

Trait	ZH8015	*es4*
Plant height/cm	109.9 ± 2.33	81 ± 2.11^**^
Number of productive panicles per plant	14.2 ± 2.43	14.1 ± 0.38
Number of spikelets per panicle	106.13 ± 11.76	89.49 ± 5.04^**^
Seed setting rate/%	85.47 ± 0.04	77.39 ± 0.02^*^
1000-grain weight/g	37.45 ± 0.82	34.22 ± 0.92^**^

*^∗^Significance at P < 0.05, ^∗∗^ extremely significance at P < 0.01.*

**FIGURE 2:**
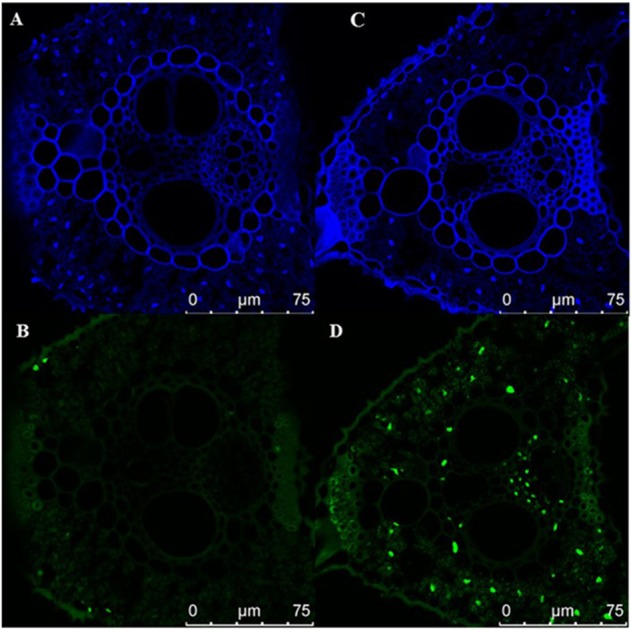
TUNEL assay the cell death. **(A,B)** ZH8015. **(C,D)** *es4*. Blue signal is DAPI staining, green color represents positive result. Scale bar = 75 μm.

Early senescence usually induces the accumulation of H_2_O_2_ in rice leaves. These toxic ROS can further result in lipid peroxidation, cellular damage and cell death. DAB staining showed that the color of es4 leaves is darker than those in ZH8015 as a result of more accumulation of H_2_O_2_ in early senescence leaves of *es4* (Figure 1C). Evans blue staining is an indicator of irreversible membrane damage or cell death. After Evans blue staining, *es4* exhibited a deep blue at the site of necrosis, however, leaves of ZH8015 exhibited slight blue (Figure 1D), indicating that there was more cell death in *es4*. Early senescence often leads more accumulation of Superoxide radicals concomitantly. As an indicator of Superoxide radicals’ accumulation, the results of NBT staining indicated that more blue formazan precipitates appeared in *es4* leaves (Figure 1E). Therefore, there was more Superoxide radicals accumulation in *es4*. To confirm that there are more cell deaths in *es4*, the third leaves of *es4* mutant and ZH8014 at 70 dps were subjected to a TUNEL assay. Few of the nuclei in leaf sections of ZH8015 were TUNEL positive, whereas numerous nuclei in leaf sections of *es4* were TUNEL positive (Figure 2). The results of TUNEL assays indicated that there were more cell apoptosis in *es4*. We also analyzed the expression levels of some senescence-related genes, and the results indicated that the expression levels of senescence-related genes including *OsI57*, *OsI85,* and *OsH36* were significantly upregulated in *es4*, but that *OsAPX1* was significantly down regulated in *es4* (Figure 1G). These results indicated that the mutation of *ES4* generally induced accumulation of ROS, DNA damage and accelerated cell senescence in *es4* leaves.

### Alteration of Photosynthetic Ability, Chlorophyll Content and Chloroplast Ultrastructure

**FIGURE 3:**
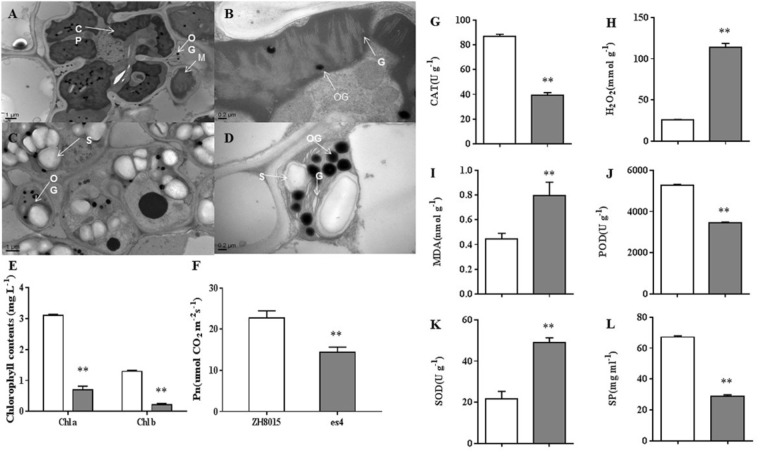
Ultrastructure of the chloroplast; senescence related-indices of ZH8015 and *es4* at 70 dps. Photosynthetic ability analysis in flag leaves of ZH8015 and *es4* at 70 dps. Senescence related-indices of ZH8015 and *es4* at 70 dps. **(A,C)** Ultrastructure of the chloroplast in the flag leaf of ZH8015 at 70 dps. **(B,D)** Ultrastructure of the chloroplast in the flag leaf of *es4* at 70 dps. CP, chloroplast; G, granum; M, mitochondrion; OG, osmiophilic granule; S, starch granule. Scale bar = 0.2 μm. **(E)** Content of chlorophyll a and b. **(F)** Net photosynthetic rate (*P*_n_). **(G)** The enzymatic activities of catalase (CAT). **(H)** The content of H_2_O_2_. **(I)** The content of malondialdehyde (MDA). **(J)** The enzymatic activities of peroxidase (POD). **(K)** The enzymatic activities of superoxide dismutase (SOD). **(L)** The content of soluble proteins (SP) (mean ± SD, *n* = 3), ^∗^ significance at *P* < 0.05, ^∗∗^extremely significance at *P* < 0.01.

*P*_n_, content of Chlorophyll a and b were examined at 70 dps in ZH8015 and *es4*. Compared to the wild-type plants, the *es4* mutant’s *P*_n_, content of chlorophyll a and b was only 63.52, 22.45, and 16.54%, respectively (Figure 3E,F). These results indicated that *es4* mutant exhibited reduction in net photosynthetic rate and content of chlorophyll a and b.

Transmission electron microscopy (TEM) analysis revealed that the number and size of chloroplasts were dramatically reduced in third leaves of *es4* mutants compared to the leaves of wild-type plants. The cell of ZH8015 leaves exhibited integrated chloroplast membrane and orderly stroma lamellae structure (Figure 3A,B). Conversely, some chloroplasts membranes were dissolved and the thylakoids were disorderly arranged or degraded in *es4* mutant leaves, and we also observed more osmiophilic granules and starch grains in *es4* (Figure 3C,D). All the results indicated that the mutation of *ES4* may lead to abnormal chloroplast development.

### Determination of Physiological Parameters Related to Senescence

We measured the content of senescence-related substance, including H_2_O_2_ and MDA, and the activities of CAT, POD, and SOD in the third leaves of ZH8015 and *es4* mutant, respectively. The results showed that content of H_2_O_2_ and MDA were remarkably higher in *es4* leaves than that in ZH8015. The activities of CAT, POD and the content of SP were decreased in *es4*, while the activity of SOD increased in *es4* mutant (Figure 3G–L). Therefore, the mutation of *ES4* might lead to accumulation of H_2_O_2_.

### Genetic Analysis, Mapping and Function Analysis of *ES4*

To isolate the premature leaf senescence gene responsible for the *es4* mutant phenotype, we crossed the *es4* mutant with 02428 to generate F_1_ population. All F_1_ individuals from the cross *es4*/02428 showed the normal green phenotype similar with ZH8015. In the F_2_ segregating population, 1634 normal plants and 580 early senescent plants showed a typical segregation ratio of 3:1(*x*^2^= 1.69 < *x*^2^_0.05_ = 3.84, Supplementary Table S1). These results suggested that the early senescence phenotype was controlled by a single recessive nuclear gene.

**FIGURE 4:**
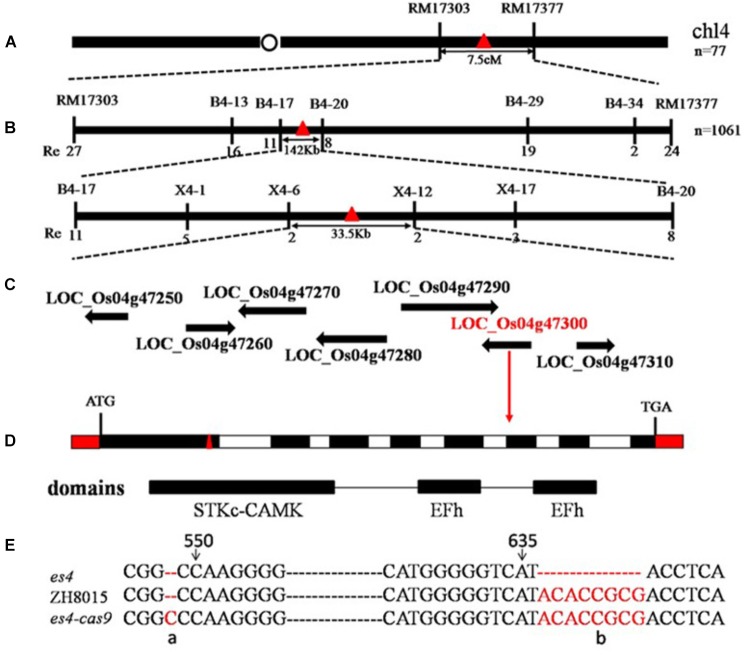
Map-based cloning of *es4*. **(A)** Location of *es4* on chromosome. **(B)** A total of 1061 mutant type F_2_ individuals were used for fine mapping. **(C)** Seven ORF were found. **(D)** *ES4* gene structure and CRISPR/Cas9 site of *es4-cas9*. **(E)** Mutant site of *es4* and CRISPR/Cas9 site of *es4-cas9*.

1061 early senescent plants in F_2_ were used to locate *ES4* by map-based cloning. *ES4* was primarily mapped in the region linked to markers RM17303 and RM17377 on the long arm of chromosome 4 and subsequently fine mapped between X4–6 and X4–12 with a 33.5 Kb physical distance (Figure 4A,B). Seven open reading frames were annotated in this region according to the Rice Genome Annotation Project^3^. The genome DNA sequencing analysis of the final region revealed eight bases deletion at position 636 to 643 bp in the first exon of the sixth ORF named *LOC_Os04g47300* encoding a Calcium-dependent protein kinase (*OsCPK12*) (Figure 4E). The *es4* mutation led to a frameshift mutation in the STKc_CAMK domain of *LOC_Os04g47300* (Figure 4C,D).

**FIGURE 5:**
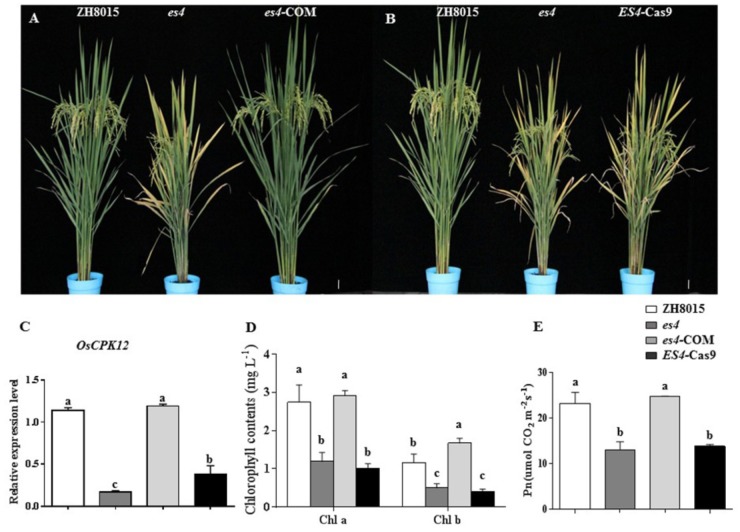
Functional complementation and CRISPR/Cas9 knock-out of *ES4*. **(A)** Plant of ZH8015, *es4*, and *es4-COM* at the mature stage. **(B)** Plant of ZH8015, *es4*, and *es4-cas9* at the mature stage. **(C)** *OsCPK12* relative expression level in ZH8015, *es4*, *es4-COM*, and *ES4-cas9*. **(D)** Content of Chlorophyll a and b in flag leaf of ZH8015, *es4*, *es4-COM*, and *ES4-cas9* at the tillering stage. **(E)** Net photosynthetic rate (*P*_n_) in flag leaf of ZH8015, *es4*, *es4-COM*, and *ES4-cas9* at 70 dps (mean ± SD, *n* = 3), Scale bar = 4 cm. Letters in the figure indicate the results of multiple comparison test, a,b,c indicate significant difference on 0.01 level.

To confirm that the *OsCPK12* mutant was indeed responsible for the *es4* mutant phenotype, the complementary vector pCAMBIA1300-*OsCPK12* was transformed into the *es4* calli. All the transgenic plants (*es4*-COM) restored to the wild-type phenotype (Figure 5A), and content of chlorophyll a and b, the expression level of *OsCPK12* and *P*_n_ were also restored to wild-type levels (Figure 5C–E). Furthermore, the CRISPR/cas9 vector Cas9/gRNA was constructed to knock out the *ES4* gene. *ES4*-Cas9 had a cytosine insertion at position 548–549 bp (Figure 4E) and all the transgenic plants (*ES4*-Cas9) exhibited the premature senescence (Figure 5B). Content of chlorophyll a and b, the expression levels of *OsCPK12* and *P*_n_ were significantly reduced compared with ZH8015 (Figure 5C–E). These results demonstrated that the frameshift mutation of *OsCPK12* was responsible for premature senescence phenotype in *es4*.

### Enhancement of *P*_n_ and Content of Chlorophylls in Overexpression Plants of *OsCPK12*

**FIGURE 6:**
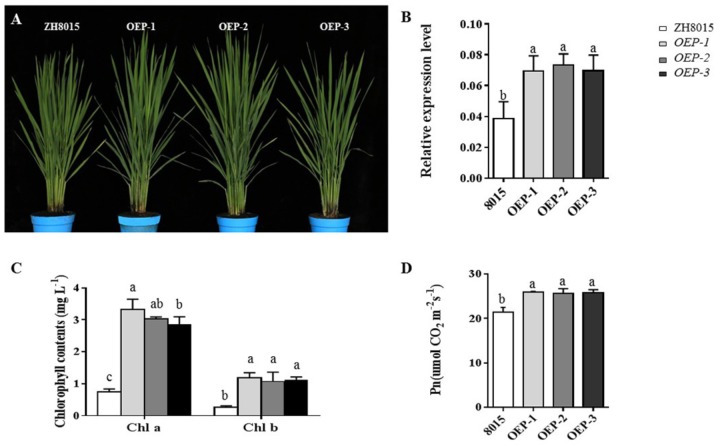
Phenotypic characteristics of ZH8015 and overexpression plants OEP-1, OEP-2, and OEP-3; the relative expression level of *OsCPK12* in ZH8015 and overexpression plants OEP-1, OEP-2, and OEP-3; the content of Chlorophyll a and b and *P*_n_. **(A)** Plant of ZH8015 and *OsCPK12*-OEP at 70 dps. content. **(B)** The expression of *OsCPK12*. **(C)** The content of Chlorophyll a and b. **(D)** Net photosynthetic rate (*P*_n_) (mean ± SD, *n* = 3), scale bar = 4 cm. Letters in the figure indicate the results of multiple comparison test, a,b,c indicate significant difference on 0.05 level.

To further verify the function of *OsCPK12*, the overexpression plants (OEPs) were constructed (Figure 6A). We found that the expression level of *OsCPK12* in the overexpression transgenic lines was approximately 1.83-fold higher than that in ZH8015 (Figure 6B). The *P*_n_ and content of chlorophyll a and b in *ES4*-OEP plants were also higher than that in wide-type plants (Figure 6C,D). Therefore, the overexpression of *OsCPK12* might enhance the *P*_n_ and content of Chlorophyll a and b, and thus leading to the delay of the growth period in rice.

### The Expression Pattern of *ES4*

**FIGURE 7:**
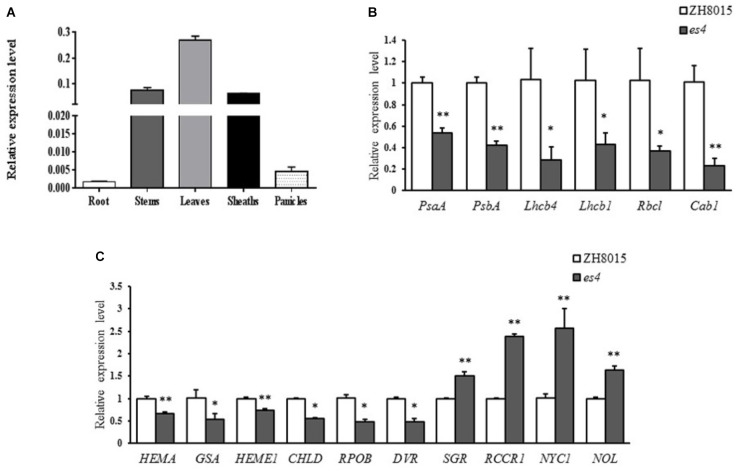
qRT-PCR analysis of *OsCPK12*, some photosynthesis and chlorophyll synthesis related genes. **(A)** Expression levels of *OsCPK12* in different organs. **(B)** Expression levels of photosynthesis related genes. **(C)** Expression levels of synthesis and degradation of chlorophyll related genes (mean ± SD, *n* = 3), ^∗^ significance at *P* < 0.05, ^∗∗^ extremely significance at *P* < 0.01.

To determine the expression pattern of *ES4* in rice, qRT-PCR was conducted using *ES4* specific primers. As expected, the expression level in leaves was much higher than any other organs because the senescence phenotype appeared on leaves mainly. The expression levels in stems and sheaths were also higher while the expression levels were much lower in roots and panicles. The results revealed that although *ES4* was ubiquitously expressed in many organs, and it was mainly expressed in photosynthetic organs such as stems and sheaths, especially in leaves (Figure 7A).

### Expression of Some Photosynthesis and Chlorophyll Synthesis Related Genes

To understand the molecular basis of early senescence and photosynthesis and synthesis in *es4*, the relative expression levels of photosynthesis and synthesis-associated genes including *OsPsaA*, *OsPsbA*, *OsLhcb1*, *OsLhcb4*, *OsCab1*, *OsRbcl, Hema*, *GSA*, *Heme1*, *Chld*, *RpoB*, *DVR*, *SGR*, *Rccr1*, *NYC1*, and *NOL* were examined in ZH8015 and *es4* by qRT-PCR at 70 dps. The expression levels of synthesis related genes including *Hema*, *Heme1*, *GSA*, *Chld*, *RpoA*, and *DVR* (Goslings et al., 2004; Zhang et al., 2006; Wang et al., 2010; Wang and Deng, 2013; Ohmiya et al., 2014) were significantly down-regulated in *es4* (Figure 7B) and the expression levels of chlorophyll degradation related genes including *SGR*, *Rccr1*, *NYC1*, and *NOL* (Jiang et al., 2007; Kusaba et al., 2007; Tang et al., 2011; Sakuraba et al., 2013) were significantly up-regulated in *es4* (Figure 7A). Photosynthesis related genes including *OsPsaA*, *OsPsbA*, *OsLhcb1*, *OsLhcb4*, *OsCab1*, and *OsRbcl* (Caffarri et al., 2004; Mei et al., 2017) were significantly down-regulated in *es4* (Figure 7C). The results indicated that the mutation of *ES4* was responsible for the decline of photosynthetic ability and the content of chlorophylls in *es4*.

## Discussion

During the process of plant senescence, the leaves undergo a series of physical and physiological changes: the breakdown of chloroplasts, the degradation of chlorophyll and the change of leaves color from green to yellow, the decrease in content of proteins (soluble protein especially), rise in the content and activity of hydrolases, enhancement of the content of MDA, the decreased activities of free radicals and active oxygen scavenging enzymes (such as SOD, POD, and CAT), and cell death (Wittenbach, 1977; Hua and Wang, 2003). Senescence is also associated with an increased production of ROS such as H_2_O_2_, superoxide and toxic derivative hydroxyl radical (Van Breusegem and Dat, 2006). These toxic ROS can result in lipid peroxidation, cellular damage and cell death, and genetic evidence suggests that ROS as a signaling molecule plays a major role in the senescence process by genetically activating programmed pathways of gene expression (Foyer and Noctor, 2005). These studies have established some aging indicators and deepened our understanding of leaf senescent process. In this study, we isolated an *es4* mutant from ZH8015 characterized by an early senescence phenotype. *es4* exhibited the breakdown of chloroplasts, the reduction of chlorophyll content, photosynthetic rate and crop yield. The activities of CAT, POD and the level of SP in *es4* were significantly lower than those of ZH8015, while the content of H_2_O_2_, Superoxide radicals and MDA were significantly higher than those of ZH8015. These results were consistent with those found in other early senescence mutants in rice. Therefore, *es4* is determined an early senescence mutant. SOD, as a biocatalyst, plays an important role in the aging and defense process of plants by disproportionating O_2_^⋅-^ into H_2_O_2_ and O_2_. In some previous studies the change of SOD activity is unstable. For example, during the senescence of three representative cold super rice varieties, the SOD activity firstly increased and then declined (Yin et al., 2009). Other studies showed the leaf senescence is relevant to the reduction of SOD activity (Hua and Wang, 2003). Some studies showed the activity of SOD was increased in the mutant with senescent phenotype such as *spl32* (Sun et al., 2017). In our study, the activities of SOD in *es4* were significantly increased. The difference in SOD activity may be related with different measurement period in different studies. Our samples were taken from ZH8015 and *es4* at 70 dps. but during this period, the lower leaves of *es4* became yellow. O_2_^⋅-^ is a necessary production of photooxidation during leaf senescence, and O_2_^⋅-^ increased sharply during this stage. However, SOD is one of the systems for scavenging O_2_^⋅-^. In this stage, SOD system was not destroyed totally, so the activity of SOD increased to remove the elevated O_2_^⋅-^ content. As alternative explanations can be made for the accumulation of O_2_^⋅-^, the increased activity of SOD was still not enough to scavenge the sharply elevated O_2_^⋅-^. Therefore, the activity of SOD was significantly increased and content of O_2_^⋅-^ was also increased in *es4*. The elevation of ROS (such as O_2_^⋅-^ and H_2_O_2_)content further accelerated leaf senescence in *es4*.

Our results showed that the mutant of *OsCPK12* was responsible for the leaf early senescence of *es4*. *OsCPK12* encodes a Calcium-dependent protein kinase (CPK). CPKs participate in numerous aspects of plant growth and development; however, there are few reports about the functions of CPKs in the process of senescence. The transient expression of the constitutively active mutant of *NtCDPK2*, A CPK from tobacco, in *Nicotiana benthamian*a leaves induced ROS production, defense genes, and HR-like cell death against additional abiotic stresses (Kobayashi et al., 2007). Potato (*Solanum tuberosum*) calcium-dependent protein kinase (StCDPK5) has been shown to phosphorylate the N-terminal region of plasma membrane RBOH (respiratory burst oxidase homolog) protein and participate in StRBOHB-mediated ROS burst (Kobayashi et al., 2012). The expression of *AtCPK1*, a CPK gene in Arabidopsis, in *Rubia cordifolia* cells caused moderate and stable elevation of intracellular reactive oxygen species (ROS) levels (Bulgakov et al., 2011). Ectopic expression of *OsCPK13* (*OsCDPK7*) in rice without abnormal phenotype led to improved abiotic stress tolerance (Saijo et al., 2001). Moreover, expression of the rice *CDPK-7* in sorghum led to enhanced accumulation of cell death and PR proteins and elevated transcription level of some defense genes and induced a lesion mimic phenotype (Mall et al., 2011). These studies indicated that CPKs may involve in regulating senescence directly or indirectly by mediating the content of ABA and ROS in plants. OsCPK12 is a member of the CPKs family in rice with functions in multiple signaling pathways. Ye’s study indicated that the expression level of *OsCPK12* was up-regulated in the endosperm stage (Ye et al., 2009). *OsCPK12* also oppositely modulates salt-stress tolerance and blast disease resistance for the decrease of ROS content in overexpression of *OsCPK12* plants (Asano et al., 2012). Besides, the Arabidopsis CPK12 was a negative ABA-signaling regulator in seed germination and post-germination growth (Zhao et al., 2011). *OsCPK12* involved in the signal transduction pathways and the overexpression of *OsCPK12* demonstrated increased ability to growth under low nitrogen conditions (Asano et al., 2012). *OsCPK12* was involved in nitrogen metabolism and overexpression of *OsCPK12* increased nitrogen-use efficiency, improving yields when little nitrogen was available (Xing et al., 2018). Xing’s studies have confirmed that *OsCPK12* is indeed related to premature senescence of rice. Researchers cloned a gene namely ESL4 about rice leaf senescence which was the same as ES4. The *esl4* mutant became yellow at the early tillering stage, and the senescent phenotype was developed gradually at an early stage of heading. Their results indicated *OsCPK12* was involved in nitrogen metabolism thus resulted in leaf senescence (Xing et al., 2018). However, our study found that *OsCPK12* was not only related with nitrogen metabolism but also related with chlorophyll metabolism and photosynthesis.

In our study, the *P*_n_ and content of Chlorophyll a and b in *es4* and ES4-cas9 were significantly lower than those in ZH8015. In the complementary transgenic plants, *P*_n_ and content of Chlorophyll a and b were the same as those in ZH8015. The overexpression of *OsCPK12* enhanced the *P*_n_ and content of chlorophylls in *ES4*-OEP plants. Overexpression of *OsCPK12* in ZH8015 also resulted in a delayed leaf senescence. Compared with ZH8015, the expression levels of five photosynthesis related genes were down-regulated in *es4*; the expression levels of Chlorophyll synthesis related genes slightly down-regulated in *es4* and the expression levels of Chlorophyll degradation related genes slightly up-regulated in *es4*. These findings suggested that the *OsCPK12* also participates in both chlorophyll metabolism and photosynthesis. Previous studies have shown that chlorophyll content had a positive and significant correlation with yield in rice (Ghosh et al., 2003). Leaf photosynthesis in rice was also related to grain yield (Ishii, 1993). The grain yield of rice is directly determined by the number of panicles per plant, the number of grains per panicle, and grain weight (Huo et al., 2017). There was no significant difference in the number of panicles per plant between ZH8015 and *es4*, but the spikelet number per panicle, seed setting rate and 1000-grain weight of *es4* were lower than those of ZH8015, which resulted in a poor yield in *es4*. Therefore, in our study, leaf photosynthesis and chlorophyll content also had a positive relation with grain yield. The mutant of *OsCPK12* severely reduced rice yield. Taken together, our results showed that *OsCPK12* function is multidimensional and that it plays a very important role in the process of growth and development.

In summary, the functional loss of *OsCPK12* results in the changes in activities of CAT, POD, and SOD, accumulation of the ROS and MDA, reduction of *P*_n_ and chlorophyll content, which eventually leads to leaf senescence and reduced yields. These results suggest that *OsCPK12* not only plays an important role in biotic and abiotic stress, nitrogen metabolism but also involves the process of leaf senescence in rice, and the overexpression of *OsCPK12* might provide the potential raise on productivity in rice.

## Author Contributions

BW performed most of the research and drafted the manuscript. YZ designed the experiments. ZB carried out the nucleotide polymorphism analysis. QL, TX, NY, YC, and AZ analyzed the data. QL, TX, WW, XZ, GA, PY, and DC revised the manuscript. SC and LC supervised the study and revised the manuscript. All authors read and approved the final manuscript.

## Conflict of Interest Statement

The authors declare that the research was conducted in the absence of any commercial or financial relationships that could be construed as a potential conflict of interest.

## Footnotes

1 http://gramene.org/genome_browser/index.html

2 http://cbi.hzau.edu.cn/cgi-bin/CRISPR

3 http://rice.plantbiology.msu.edu/

## References

[B1] Alonso J. M. Hirayama T. Roman G. Nourizadeh S. Ecker J. R. (1999). EIN2, a bifunctional transducer of ethylene and stress responses in Arabidopsis. *Science* 284:2148. 10.1126/science.284.5423.2148 10381874

[B2] Asano T. Hakata M. Nakamura H. Aoki N. Komatsu S. Ichikawa H. (2011). Functional characterisation of OsCPK21, a calcium-dependent protein kinase that confers salt tolerance in rice. *Plant Mol. Biol.* 75 179–191. 10.1007/s11103-010-9717-1 21136139

[B3] Asano T. Hayashi N. Kobayashi M. Aoki N. Miyao A. Mitsuhara I. (2012). A rice calcium-dependent protein kinase OsCPK12 oppositely modulates salt-stress tolerance and blast disease resistance. *Plant J.* 69 26–36. 10.1111/j.1365-313X.2011.04766.x 21883553

[B4] Asano T. Kunieda N. Omura Y. Ibe H. Kawasaki T. Takano M. (2002). Rice SPK, a calmodulin-like domain protein kinase, is required for storage product accumulation during seed development phosphorylation of sucrose synthase is a possible factor. *Plant Cell* 14 619–628. 10.1105/tpc.010454 11910009 PMC150584

[B5] Berkowitz G. Zhang X. Mercier R. Leng Q. Lawton M. (2000). Co-expression of calcium-dependent protein kinase with the inward rectified guard cell K+ channel KAT1 alters current parameters in *Xenopus laevis* oocytes. *Plant Cell Physiol.* 41 785–790. 10.1093/pcp/41.6.785 10945349

[B6] Bulgakov V. P. Gorpenchenko T. Y. Shkryl Y. N. Veremeichik G. N. Mischenko N. P. Avramenko T. V. (2011). CDPK-driven changes in the intracellular ROS level and plant secondary metabolism. *Bioeng. Bugs* 2 327–330. 10.4161/bbug.2.6.16803 22064507

[B7] Caffarri S. Croce R. Cattivelli L. Bassi R. (2004). A look within LHCII: differential analysis of the Lhcb1-3 complexes building the major trimeric antenna complex of higher-plant photosynthesis. *Biochemistry* 43:9467. 10.1021/bi036265i 15260489

[B8] Campo S. Baldrich P. Messeguer J. Lalanne E. Coca M. San Segundo B. (2014). Overexpression of a calcium-dependent protein kinase confers salt and drought tolerance in rice by preventing membrane lipid peroxidation. *Plant Physiol.* 165 688–704. 10.1104/pp.113.230268 24784760 PMC4044838

[B9] Chen Y. Xu Y. Luo W. Li W. Chen N. Zhang D. (2013). The F-box protein OsFBK12 targets OsSAMS1 for degradation and affects pleiotropic phenotypes, including leaf senescence, in rice. *Plant Physiol.* 163 1673–1685. 10.1104/pp.113.224527 24144792 PMC3850201

[B10] Cheng S. H. Willmann M. R. Chen H. C. Sheen J. (2002). Calcium signaling through protein kinases. The Arabidopsis calcium-dependent protein kinase gene family. *Plant Physiol.* 129 469–485. 10.1104/pp.005645 12068094 PMC1540234

[B11] Chung H. J. Sehnke P. C. Ferl R. J. (1999). The 14-3-3 proteins: cellular regulators of plant metabolism. *Trends Plant Sci.* 4 367–371. 10.1016/S1360-1385(99)01462-4 10462770

[B12] El Mannai Y. Akabane K. Hiratsu K. Satoh-Nagasawa N. Wabiko H. (2017). The NAC transcription factor gene *OsY37* (ONAC011) promotes leaf senescence and accelerates heading time in rice. *Int. J. Mol. Sci.* 18:E2165. 10.3390/ijms18102165 29039754 PMC5666846

[B13] Fanata W. I. Lee K. H. Son B. H. Yoo J. Y. Harmoko R. Ko K. S. (2013). N-glycan maturation is crucial for cytokinin-mediated development and cellulose synthesis in *Oryza sativa*. *Plant J.* 73 966–979. 10.1111/tpj.12087 23199012

[B14] Foyer C. H. Noctor G. (2005). Redox homeostasis and antioxidant signaling: a metabolic interface between stress perception and physiological responses. *Plant Cell* 17 1866–1875. 10.1105/tpc.105.033589 15987996 PMC1167537

[B15] Ghosh M. Pal A. K. Pal S. K. (2003). Relationship of leaf area and chlorophyll content with yield of aromatic rice. *Indian J. Plant Physiol.* 8 199–200.

[B16] Goslings D. Meskauskiene R. Kim C. Lee K. P. Nater M. Apel K. (2004). Concurrent interactions of heme and FLU with Glu tRNA reductase (HEMA1), the target of metabolic feedback inhibition of tetrapyrrole biosynthesis, in dark- and light-grown Arabidopsis plants. *Plant J.* 40 957–967. 10.1111/j.1365-313X.2004.02262.x 15584960

[B17] Harmon A. C. Gribskov M. Gubrium E. Harper J. F. (2001). The CDPK superfamily of protein kinases. *New Phytol.* 151 175–183. 10.1046/j.1469-8137.2001.00171.x 33873379

[B18] He Y. Li L. Zhang Z. Wu J. L. (2018). Identification and comparative analysis of premature senescence leaf mutants in rice (*Oryza sativa* L.). *Int. J. Mol. Sci.* 19:E140. 10.3390/ijms19010140 29301377 PMC5796089

[B19] Himelblau E. (2000). Molecular aspects of leaf senescence. *Trends Plant Sci.* 5 278–282. 10.1016/S1360-1385(00)01655-1 10871899

[B20] Hrabak E. M. Chan C. W. Gribskov M. Harper J. F. Choi J. H. Halford N. (2003). The Arabidopsis CDPK-SnRK superfamily of protein kinases. *Plant Physiol.* 132 666–680. 10.1104/pp.102.011999 12805596 PMC167006

[B21] Hua C. Wang R. (2003). Changes of SOD and CAT activities and MDA content during senescence of hybrid rice and three lines leaves. *Acta Botanica Boreali Occidentalia Sinica* 23 406–409.

[B22] Huo X. Wu S. Zhu Z. Liu F. Fu Y. Cai H. (2017). *NOG1* increases grain production in rice. *Nat. Commun.* 8:1497. 10.1038/s41467-017-01501-8 29133783 PMC5684330

[B23] Ishii R. (1993). “Leaf photosynthesis in rice in relation to grain yield,” in *Photosynthesis: Photoreactions to Plant Productivity*, eds Abrol Y. P. Mohanty P., and Govindjee (Dordrecht: Kluwer Academic Publishers), 561–569. 10.1007/978-94-011-2708-0_24

[B24] Jiang H. Li M. Liang N. Yan H. Wei Y. Xu X. (2007). Molecular cloning and function analysis of the stay green gene in rice. *Plant J.* 52 197–209. 10.1111/j.1365-313X.2007.03221.x 17714430

[B25] Jiao B. Wang J. Zhu X. Zeng L. Li Q. He Z. (2012). A novel protein RLS1 with NB-ARM domains is involved in chloroplast degradation during leaf senescence in rice. *Mol. Plant* 5 205–217. 10.1093/mp/ssr081 21980143

[B26] Kang K. Kim Y. S. Park S. Back K. (2009). Senescence-induced serotonin biosynthesis and its role in delaying senescence in rice leaves. *Plant Physiol.* 150 1380–1393. 10.1104/pp.109.138552 19439571 PMC2705024

[B27] Kobayashi M. Ohura I. Kawakita K. Yokota N. Fujiwara M. Shimamoto K. (2007). Calcium-dependent protein kinases regulate the production of reactive oxygen species by potato NADPH oxidase. *Plant Cell* 19 1065–1080. 10.1105/tpc.106.048884 17400895 PMC1867354

[B28] Kobayashi M. Yoshioka M. Asai S. Nomura H. Kuchimura K. Mori H. (2012). StCDPK5 confers resistance to late blight pathogen but increases susceptibility to early blight pathogen in potato via reactive oxygen species burst. *New Phytol.* 196 223–237. 10.1111/j.1469-8137.2012.04226.x 22783903

[B29] Kong X. Li D. (2011). Hydrogen peroxide is not involved in HrpN from *Erwinia amylovora*-induced hypersensitive cell death in maize leaves. *Plant Cell Rep.* 30 1273–1279. 10.1007/s00299-011-1038-6 21344189

[B30] Kusaba M. Ito H. Morita R. Iida S. Sato Y. Fujimoto M. (2007). Rice NON-YELLOW Coloring1 is involved in light-harvesting complex II and grana degradation during leaf senescence. *Plant Cell* 19 1362–1375. 10.1105/tpc.106.042911 17416733 PMC1913755

[B31] Kusumi K. Sakata C. Nakamura T. Kawasaki S. Yoshimura A. Iba K. (2011). A plastid protein NUS1 is essential for build-up of the genetic system for early chloroplast development under cold stress conditions. *Plant J.* 68 1039–1050. 10.1111/j.1365-313X.2011.04755.x 21981410

[B32] Lei Z. Chen J. Chen X. (2007). The physiological functions of calcium-dependent protein kinases in plant calcium signal transduction. *J. Fujian Forestry Sci. Technol.* 34 244–249. 10.3969/j.issn.1002-7351.2007.03.059

[B33] Li J. Lee Y. R. J. Assmann S. M. (1998). Guard cells possess a calcium-dependent protein kinase that phosphorylates the KAT1 potassium channel. *Plant Physiol.* 116 785–795. 10.1104/pp.116.2.785 9489023 PMC35138

[B34] Li M. Li X. Zhou Z. Wu P. Fang M. Pan X. (2016). Reassessment of the four yield-related genes Gn1a, DEP1, GS3, and IPA1 in rice using a CRISPR/Cas9 system. *Front. Plant Sci.* 7:377. 10.3389/fpls.2016.00377 27066031 PMC4811884

[B35] Li Z. Peng J. Wen X. Guo H. (2013). Ethylene-insensitive3 is a senescence-associated gene that accelerates age-dependent leaf senescence by directly repressing miR164 transcription in Arabidopsis. *Plant Cell* 25 3311–3328. 10.1105/tpc.113.113340 24064769 PMC3809534

[B36] Mall T. K. Dweikat I. Sato S. J. Neresian N. Xu K. Ge Z. (2011). Expression of the rice CDPK-7 in sorghum: molecular and phenotypic analyses. *Plant Mol. Biol.* 75 467–479. 10.1007/s11103-011-9741-9 21318369

[B37] Mei J. Li F. Liu X. Hu G. Fu Y. Liu W. (2017). Newly identified *CSP41b* gene localized in chloroplasts affects leaf color in rice. *Plant Sci.* 256 39–45. 10.1016/j.plantsci.2016.12.005 28167036

[B38] Murry M. G. Thompson W. F. (1980). Rapid isolation of high molecular weight plant DNA. *Nucleic Acids Res.* 8:4321 10.1093/nar/8.19.4321 PMC324241 7433111

[B39] Ohmiya A. Hirashima M. Yagi M. Tanase K. Yamamizo C. (2014). Identification of genes associated with chlorophyll accumulation in flower petals. *PLoS One* 9:e113738. 10.1371/journal.pone.0113738 25470367 PMC4254739

[B40] Pei Z. M. Ward J. M. Harper J. F. Schroeder J. I. (1996). A novel chloride channel in Vicia faba guard cell vacuoles activated by the serine/threonine kinase, CDPK. *EMBO J.* 15 6564–6574. 10.1002/j.1460-2075.1996.tb01047.x 8978683 PMC452481

[B41] Porra R. J. Schäfer W. Cmiel E. Katheder I. Scheer H. (1994). The derivation of the formyl-group oxygen of chlorophyll b in higher plants from molecular oxygen. Achievement of high enrichment of the 7-formyl-group oxygen from 18O2 in greening maize leaves. *Eur. J. Biochem.* 219 671–679. 10.1111/j.1432-1033.1994.tb19983x 8307032

[B42] Putnam E. C. Harmon A. C. Palevitz B. A. Fechheimer M. Cormier M. J. (1989). Calcium dependent protein kinase is localized with F-actin in plant cells. *Cell Motil. Cytoskel.* 1 12–22. 10.1002/cm.970120103

[B43] Ramel F. Sulmon C. Bogard M. Couée I. Gouesbet G. (2009). Differential patterns of reactive oxygen species and antioxidative mechanisms during atrazine injury and sucrose-induced tolerance in *Arabidopsis thaliana* plantlets. *BMC Plant Biol.* 9:28. 10.1186/1471-2229-9-28 19284649 PMC2661893

[B44] Ray S. Agarwal P. Arora R. Kapoor S. Tyagi A. K. (2007). Expression analysis of calcium-dependent protein kinase gene family during reproductive development and abiotic stress conditions in rice (*Oryza sativa* L. ssp. indica). *Mol. Genet. Genom.* 278 493–505. 10.1007/s00438-007-0267-4 17636330

[B45] RH L. MC L. SC C. (2004). A novel alkaline alpha-galactosidase gene is involved in rice leaf senescence. *Plant Mol. Biol.* 55 281–295. 10.1007/s11103-004-0641-0 15604681

[B46] Saijo Y. Kinoshita N. Ishiyama K. Hata S. Kyozuka J. Hayakawa T. (2001). A Ca^2+^-dependent protein kinase that endows rice plants with cold- and salt-stress tolerance functions in vascular bundles. *Plant Cell Physiol.* 42 1228–1233. 10.1093/pcp/pce158 11726707

[B47] Sakuraba Y. Kim Y. S. Yoo S. C. Peak N. C. (2013). 7-Hydroxymethyl chlorophyll a reductase functions in metabolic channeling of chlorophyll breakdown intermediates during leaf senescence. *Biochem. Biophys. Res. Commun.* 430 32–37. 10.1016/j.bbrc.2012.11.050 23200839

[B48] Simeunovic A. Mair A. Wurzinger B. Teige M. (2016). Know where your clients are: subcellular localization and targets of calcium-dependent protein kinases. *J. Exp. Bot.* 67:3855. 10.1093/jxb/erw157 27117335

[B49] Singh S. Giri M. K. Singh P. K. Siddiqui A. Nandi A. K. (2013). Down-regulation of OsSAG12-1 results in enhanced senescence and pathogen-induced cell death in transgenic rice plants. *J. Biosci.* 38 583–592. 10.1007/s12038-013-9334-7 23938390

[B50] Sun L. Wang Y. Liu L. Wang C. Gan T. Zhang Z. (2017). Isolation and characterization of a spotted leaf 32 mutant with early leaf senescence and enhanced defense response in rice. *Sci. Rep.* 7:41846. 10.1038/srep41846 28139777 PMC5282590

[B51] Tang Y. Li M. Chen Y. Wu P. Wu G. Jiang H. (2011). Knockdown of OsPAO and OsRCCR1 cause different plant death phenotypes in rice. *J. Plant Physiol.* 168 1952–1959. 10.1016/j.jplph.2011.05.026 21807436

[B52] Thordal-Christensen H. Zhang Z. Wei Y. Collinge D. B. (1997). Subcellular localization of H2O2 in plants. H2O2 accumulation in papillae and hypersensitive response during the barley-powdery mildew interaction. *Plant J.* 11 1187–1194. 10.1046/j.1365-313X.1997.11061187.x

[B53] Van Breusegem F. Dat J. F. (2006). Reactive oxygen species in plant cell death. *Plant Physiol.* 141 384–390. 10.1104/pp.106.078295 16760492 PMC1475453

[B54] Wang N. Wang Y. Zhang Z. Zhu L. Zhang R. (2001). Senescence induced changes in autophosphorylation status and catalytic activity of plasma membrane protein kinases in soybean primary leaves. *Acta Photophysiol. Sin.* 27 33–42.

[B55] Wang P. Deng X. (2013). One divinyl reductase reduces the 8-vinyl groups in various intermediates of chlorophyll biosynthesis in a given higher plant species, but the isozyme differs between species. *Plant Physiol.* 161 521–534. 10.1104/pp.112.208421 23154534 PMC3532282

[B56] Wang P. Gao J. Wan C. Zhang F. Xu Z. Huang X. (2010). Divinyl chlorophyll(ide) a can be converted to monovinyl chlorophyll(ide) a by a divinyl reductase in rice. *Plant Physiol.* 153 994–1003. 10.1104/pp.110.158477 20484022 PMC2899930

[B57] Wellburn A. R. (1994). The spectral determination of chlorophylls a and b, as well as total carotenoids, using various solvents with spectrophotometers of different resolution. *J. Plant Physiol.* 144 307–313. 10.1016/S0176-1617(11)81192-2

[B58] Wittenbach V. A. (1977). Induced senescence of intact wheat seedlings and its reversibility1. *Plant Physiol.* 59 1039–1042. 10.1104/pp.59.6.1039 16659988 PMC542501

[B59] Wu X. Kuai B. Jia J. Jing H. (2012). Regulation of leaf senescence and crop genetic improvement. *J. Integr. Plant Biol.* 54 936–952. 10.1111/jipb.12005 23131150

[B60] Xing Y. Guo S. Chen X. Du D. Liu M. Xiao Y. (2018). Nitrogen metabolism is affected in the nitrogen-deficient rice mutant esl4 with a calcium-dependent protein kinase gene mutation. *Plant Cell Physiol.* 59 2512–2525. 10.1093/pcp/pcy169 30165687

[B61] Xu J. Tian Y. Peng R. Xiong A. S. Zhu B. Jin X. F. (2010). AtCPK6, a functionally redundant and positive regulator involved in salt/drought stress tolerance in Arabidopsis. *Planta* 231:1251. 10.1007/s00425-010-1122-0 20217124

[B62] Yang S. Seo P. Yoon H. Park C. (2011). The Arabidopsis NAC transcription factor VNI2 integrates abscisic acid signals into leaf senescence via the COR/RD genes. *Plant Cell* 23 2155–2168. 10.1105/tpc.111.084913 21673078 PMC3160032

[B63] Yang X. Gong P. Li K. Huang F. Cheng F. Pan G. (2016). A single cytosine deletion in the OsPLS1 gene encoding vacuolar-type H^+^-ATPase subunit A1 leads to premature leaf senescence and seed dormancy in rice. *J. Exp. Bot.* 67 2761–2776. 10.1093/jxb/erw109 26994476 PMC4861022

[B64] Ye S. Wang L. Xie W. Wan B. Li X. Lin Y. (2009). Expression profile of calcium-dependent protein kinase (CDPKs) genes during the whole lifespan and under phytohormone treatment conditions in rice (*Oryza sativa* L. ssp. indica). *Plant Mol. Biol.* 70 311–325. 10.1007/s11103-009-9475-0 19263224

[B65] Yin C. Li C. Sun S. Zhao M. Huang Z. Zhao L. (2009). Dynamic change of SOD and POD activities of cold super rice during leaf senescence. *Crops* 3 37–39.

[B66] Zhang H. Li J. Yoo J. Yoo S. Cho S. Koh H. (2006). Rice Chlorina-1 and Chlorina-9 encode ChlD and ChlI subunits of Mg-chelatase, a key enzyme for chlorophyll synthesis and chloroplast development. *Plant Mol. Biol.* 62 325–337. 10.1007/s11103-006-9024-z 16915519

[B67] Zhao L. Shen L. Zhang W. Z. Zhang W. Wang Y. Wu W. (2013). Ca^2+^-dependent protein kinase11 and 24 modulate the activity of the inward rectifying K^+^ channels in Arabidopsis pollen tubes. *Plant Cell.* 25 649–661. 10.1105/tpc.112.103184 23449501 PMC3608784

[B68] Zhao R. Sun H. Mei C. Wang X. Yan L. Liu R. (2011). The Arabidopsis Ca^2+^-dependent protein kinase CPK12 negatively regulates abscisic acid signaling in seed germination and post-germination growth. *New Phytol.* 192 61–73. 10.1111/j.1469-8137.2011.03793.x 21692804

[B69] Zou J. Wei F. Wang C. Wu J. Ratnasekera D. Liu W. (2010). Arabidopsis calcium-dependent protein kinase CPK10 functions in abscisic acid- and Ca^2+^-mediated stomatal regulation in response to drought stress. *Plant Physiol.* 154 1232–1243. 10.1104/pp.110.157545 20805328 PMC2971602

