# Supplementary material for: Impaired Function of the Calcium-Dependent Protein Kinase, OsCPK12, Leads to Early Senescence in Rice (Oryza sativa L.)
Source: Front Plant Sci. 2019 Feb 4;10:52. doi: 10.3389/fpls.2019.00052 (PMC6369234; doi:10.3389/fpls.2019.00052)
Supplement: Supplementary file 1 [file Data_Sheet_1.docx]

**Impaired function of the** **calcium-dependent protein kinase, OsCPK12, leads to early senescence in Rice (*Oryza sativa* L.)**

**Beifang Wang^1†^, Yingxin** **Zhang^1†^, Zhenzhen Bi^1,2†^, Qunen Liu^1^, Tingting Xu^1^, Ning Yu^1^, Yongrun Cao^1^, Aike Zhu^1,3^, Weixun Wu^1^, Xiaodeng Zhan^1^,** **Galal Bakr Anis^1,4^, Ping Yu^1^, Daibo Chen^1^, Shihua Cheng^1*^, Liyong Cao^1*^**

**Table S1. Genetic analysis of mutant** ***es4* in F_2_ generation**

| Cross | F_1_ | F_2_ | | Chi-square（3：1） |
| --- | --- | --- | --- | --- |
|  |  | No. of normal plants | No. of mutant plants |  |
| 02428 / *es4* | Normal | 1634 | 580 | 1.69 |

**Table S2. Primer for vector construction in this study**

| Primer | sequence |
| --- | --- |
| ES4-COM/F | CCATGATTACGAATTCTAAGTTCAGGCTCAACAGGGT |
| ES4-COM/R | GGCCAGTGCCAAGCTTTGATTGGATAGTGAACCGACAT |
| ES4-GFP/F | CGGTCCCGGGGGATCCAAAGGAAACGAGGAGGCTTT |
| ES4-GFP/R | TGCTCACCATGGATCCGGTTTGTATTCCTTTCCTCATCATT |
| ES4-OE/F | GTAGAAGAGGTACCCGGGCACCATTCCACCCTCGCTT |
| ES4-OE/R | GCAGGTCGACTCTAGATAACTTTTTTGCGTTTCATCTGC |

**Table S3. Primer sequence used for fine mapping and qRT-PCR**

| Maker | Forward primer | Reverse primer | Purpose |
| --- | --- | --- | --- |
| B4-13 | GCAGTAACCCATAGGCACTGT | CTAGAAGTCAGCCCCGATGTT | mapping |
| B4-17 | TCGGGCAACTCACCTCCT | AAGCGGGCCTCCTCCTTT | mapping |
| B4-20 | TGGAGGTTGATCGTATATAACAG | ATGTTTTAGTGAAAGATGGGCT | mapping |
| B4-29 | TTTTTGTGTCTGTATAGGGGG | CATGAAAGATCTACTTTCTCCG | mapping |
| B4-34 | GGAGAAAGAAATGGTTGAAACA | AAGAAAAGTATTAAGGGGGCCT | mapping |
| X4-1 | CTGCTACTGCACGAAAGGATATT | AGTGGCTATATTTAGTTTGCTGC | mapping |
| X4-6 | CGCATTGTGGCTGTGTCG | GTCTGGGCGGTTTGTGTTAA | mapping |
| X4-12 | TGAAAGCAATGGATCTTTTTGA | CGCTTACCTGCATAGTTCACAT | mapping |
| X4-17 | GTAAAAAACCAAACCAGCCCA | TTAGATCGCTAAGAACACACATAT | mapping |
| *Osh36* | GCACGGAGGCGAACGA | TTGAGCGGTAGCACCCATT | qRT-PCR |
| *APX1* | AGGTGCCACAAGGAAAGATCTGGT | TCAGCAGGGCTTTGTCACTAGGAA | qRT-PCR |
| *OsI85* | GAGCAACGGCGTGGAGA | GCGGCGGTAGAGGAGATG | qRT-PCR |
| *OsI57* | ACCCTAAAGTAAATGAAGTC | CCTGCTCTTGTCTTGTTA | qRT-PCR |
| *psaA* | GCGAGCAAATAAAACACCTTTC | GTACCAGCTTAACGTGGGGAG | qRT-PCR |
| *psbA* | CCCTCATTAGCAGATTCGTTTT | ATGATTGTATTCCAGGCAGAGC | qRT-PCR |
| *Lhcb1* | CCATGTTCTCCATGTTCGGCTTCT | TAGGCCCAGGCGTTGTTGTTGA | qRT-PCR |
| *Lhcb4* | TACCTGCAGTTCGAGCTGGAC | AGGCCGAACACCTCGGTGTA | qRT-PCR |
| *RbcL* | CTTGGCAGCATTCCGAGTAA | ACAACGGGCTCGATGTGATA | qRT-PCR |
| *Cab1* | AGATGGGTTTAGTGCGACGAG | TTTGGGATCGAGGGAGTATTT | qRT-PCR |
| *Hema* | ATGGAGGCCCAAACAATCATC | GCGTAGGACCTCAGCTTCTTGA | qRT-PCR |
| *GSA* | GCTCTCCGTGACTTGACGAAAC | CCGAAGTATTCTTGAGCCCCA | qRT-PCR |
| *Heme1* | GATCCCTTGAGAACAGCAGCTG | CATTGTTAACCTCTTCCCGCAA | qRT-PCR |
| *Chld* | CGCATGCAGAATGCGAAAG | CCTCAGCAAAATCTCCACGAAA | qRT-PCR |
| *Rpoa* | CGCATGCAGGTTCATCAAAAG | CGATCAAATCTGTATTT | qRT-PCR |
| *DVR* | CGAGCCCAGGTTCATCAAGGTGC | CCTCCCGATCTTGCCGAACTCC | qRT-PCR |
| *SGR* | AGGGGTGGTACAACAAGCTG | GCTCCTTGCGGAAGATGTAG | qRT-PCR |
| *Rccr1* | CGCATTTCCTCATGGAATTT | CTTCTCACGCTGTTTGTCCA | qRT-PCR |
| *NYC1* | CATGCAACACCAACAAAAGG | GACCATTCCAGGAGAAGCAG | qRT-PCR |
| *NOL* | CCACGAAAGGTATAGGATATG | TCAAGTCAGTCACCGCAGAT | qRT-PCR |
| *OsActin1* | TGCTATGTACGTCGCCATCCAG | AATGAGTAACCACGCTCCGTCA | qRT-PCR |
